# Relationship of udder and teat conformation with intra-mammary infection in crossbred cows under hot-humid climate

**DOI:** 10.14202/vetworld.2015.898-901

**Published:** 2015-07-23

**Authors:** Pranay Bharti, Champak Bhakat, Prabhat K. Pankaj, Showkat A. Bhat, M. Arul Prakash, Mayur R. Thul, K. Puhle Japheth

**Affiliations:** 1 Livestock Production and Management Section, ICAR-National Dairy Research Institute, Karnal, Haryana, India; 2 Livestock Production and Management Section, ICAR- National Dairy Research Institute, Eastern Regional Station, Kalyani, West Bengal, India; 3 Transfer of Technology Section, ICAR- Central Research Institute for Dryland Agriculture, Hyderabad, India

**Keywords:** crossbred, somatic cell count, udder and teat conformation

## Abstract

**Aim:**

The present study was aimed to investigate the relationship of udder shape, teat-end shape, teat length, and teat diameter with intra-mammary infection in Jersey crossbred cows under hot-humid climate.

**Materials and Methods:**

A total of 24 lactating Jersey crossbred cows were evaluated for udder shape (pendulous/regular) and teat-end shape (flat/inverted/pointed) by visual examination, while teat length and teat diameter were measured using vernier caliper. Monthly milk sampling was done for 4 months of duration. Few quarters were found as blind or non-functional and so, a total of 366 quarter wise milk samples were collected at the monthly interval and subjected to somatic cell count (SCC) microscopically. The data on SCC were transformed into log scale and analyzed.

**Results:**

There was a significant (p<0.01) effect of udder shape and teat-end shape on SCC level. The mean SCC level for pendulous udder was significantly (p<0.05) higher as compared to the regular shaped udder. Similarly, significantly (p<0.05) a higher level of mean SCC was found in flat teat-end shape. A significant (p<0.01) correlation was found between SCC and teat length as well as teat diameter.

**Conclusion:**

In conclusion, pendulous udder, flat and inverted teat-end, very long and thick teat were more susceptible to intra-mammary infection in Jersey crossbred cows and these traits must be considered accordingly while selecting dairy animals for future milk production.

## Introduction

Intra-mammary infection is an imperative threat affecting the dairy sector. Udder health disorders cause profound economic loss and have a major influence on dairy cow’s welfare and productivity [1]. All the developed countries are using milk somatic cell counts (SCC) as a marker to determine the mammary health and quality of milk [2]. While most risk factors associated with management and the environment are addressed by introducing good management and hygiene measures, selecting dairy cows, which are less susceptible to mastitis is also a control measure worthy of consideration [3]. In most countries, dairy cattle breeding programs are mainly aimed at milk production traits with increasing focus on conformation traits [4]. Udder and teat conformation traits are highly heritable[5] and could serve as a marker trait for selection to reduce mastitis in dairy cattle. Some studies have revealed udder and teat conformation as risk factors for intra-mammary infection [6-7]. According to them, cows with less desirable shaped udders and more udder depth are more susceptible to lesions and contamination by mastitis-causing pathogens, which increase the risk of mastitis [7]. Identified long and thick teats are as a potential risk factor for development of mastitis. The udder and teat conformation traits were found to have a slight relation with milk somatic cell count [8] and mastitis resistance [9]. Therefore, the udder conformation traits with strong arguments can be used to improve udder health [3].

Earlier report [10] pointed out that anatomical characteristics of dairy cattle are not equal for all breeds, in a way that the udder and teat morphology could favor an individual performance or a determined breed. Few works have been done to investigate relation of udder and teat conformation with occurrence of intra-mammary infection in crossbred cattle being evolved through exotic and local breeds under different climatic conditions but information in the literature in Jersey crossbred cattle is scanty.

Therefore, the present study was carried out to evaluate the relationships of udder and teat conformation with intra-mammary infection in Jersey crossbred cows under hot-humid climate.

## Materials and Methods

### Ethical approval

The present study was carried out after getting approval by the Research Committee and Institutional Animal Ethics Committee.

### Experimental animals and location

A total of 24 lactating Jersey crossbred animals were randomly selected from the lactating herd of Eastern Regional Station, National Dairy Research Institute (ERS-NDRI), cattle yard located in Kalyani city of West Bengal. The altitude of Kalyani city is 9.75 meter above mean sea level, latitude and longitude position being 22°56′30″N and 88°32′04″E, respectively. The weather of Kalyani is hot and humid.

### Housing, feeding, and milking practices

The experimental animals were housed under loose housing system adjacent to the milking byre. The housing space of the animals was specified as per the BIS standards. The floor of the house was made up of concrete with the drainage in between covered and open space having an adequate slope for better drainage. The cleaning schedule for houses at the farm was daily in the morning from 5.30 AM to 7.00 AM and evening from 3.30 to 5.00 PM. All the feeding management practices and the feed ingredients were same as of the whole lactating herd of the farm. Concentrate and *ad libitum* green fodder was provided to complete the nutrient requirement of the lactating animals. In addition to a maintenance diet, animals were given additional concentrate at 1.0 kg for every 2.5 kg of milk produced above 5.0 kg daily yield to meet their energy requirements. Machine milking was done twice a day during the morning from 6.00 to 8.00 AM and evening from 3.00 to 5.00 PM. The milk was weighed and recorded for the individual animal.

### Evaluation and classification of udder and teat conformation

Udder shape and teat-end shape were evaluated through visual examination in the beginning of the study. Udder shape was evaluated and classified into pendulous and regular or normal udder type whereas teat-end shape was categorized as flat, inverted, and pointed. Teat length and teat diameter were measured with vernier caliper for every quarter of experimental animals in the beginning of the investigation. Teat length was taken as the distance from the base of the teat to the end of teat while the diameter of teat was measured at the mid length of the teat.

### Collection of milk samples

Approximately 30 ml of representative morning milk samples were collected aseptically in clean and sterilized sampling bottles from all the four quarters of all experimental lactating cows at monthly interval for 4 months. Udders of cows were thoroughly washed with clean water and towels soaked with antiseptic solution were used for wiping off the udder and teats and allowed to dry and first 2-3 streaks of fore milk were discarded before collection of milk samples. Few teats were found as blind or non-functional at the time of sampling and so a total of 366 quarter milk sample were collected during the whole experimental period. The collected samples were brought to the laboratory immediately for further analysis.

### Diagnosis for Intra-mammary infection

The level of intra-mammary infection was diagnosed using the microscopic method of SCC. The dried milk smear was prepared using 10 µl of milk and then stained with the modified Newman-Lampert stain for 1-2 min and then stain was drained off and smears were gently washed with tap water and then again dried. The dried stained milk smears were examined under the oil immersion lens (×100) of the microscope. Thirty different fields per smear were observed, and the average number of somatic cells per field was calculated. The average number of cells per field was then multiplied by the microscopic factor of the microscope, i.e. 240807 to obtain the number of cells per ml of the milk.

### Statistical analysis

First, the SCC were transformed into log scale to minimize the heterogeneity of variance and then classified according to various udder and teat conformation. The data were analyzed with the help of SAS software package, version 9.3 [11].

## Results

### Udder shape and SCC

The results of the present study indicated higher SCC for pendulous udder as compared to regular shaped udders. The mean ± SE of SCC for pendulous and regular shaped udders was 6.393±0.037 and 5.120±0.034, respectively (Table-1). The Analysis of Variance also showed a significant (p<0.01) effect of udders shape on the level of SCC, which has been shown in Table-2.

**Table-1:** Mean±SE of SCC in udder and teat-end shape in Jersey crossbred cows.

Traits	Number of observations	Mean±SE
Overall	366	5.377±0.039
Udder shape		
Pendulous	74	6.393±0.037^A^
Regular	292	5.120±0.034^B^
Teat-end shape		
Flat	46	6.189±0.105^a^
Inverted	90	5.850±0.068^b^
Pointed	230	5.030±0.035^c^

Mean showing different superscripts in upper case letters as well as in lower case letters in respective categories in a column differ significantly (p<0.05), SE=Standard error, SCC=Somatic cell count

**Table-2:** Analysis of variance showing the effect of udder shape and teat-end shape on SCC in Jersey crossbred cows.

Source of variance	Degree of freedom	Mean square
Udder shape	1	95.79**
Error (udder shape)	364	0.298
Teat-end shape	2	39.102**
Error (teat-end shape)	263	0.348

SCC=Somatic cell count,

** Significant (p<0.01)

### Teat-end shape and SCC

The results of the mean ± SE of SCC revealed the highest SCC for the flat teat-end shape followed by inverted and pointed teat-end. The mean ± SE of SCC for flat, inverted, and pointed teat-end shapes was 6.189±0.105, 5.850±0.068, and 5.030±0.035, respectively (Table-1), and the mean difference among flat, inverted, and pointed teat-end shape was also significant (p<0.05). Teat-end shape was also had significant (p<0.01) effect on SCC level (Table-2).

### Teat length and teat diameter

The results of teat measurements showed variation in teat length from 39.0 mm to 89.0 mm with a mean of 54.74±0.55 mm. The average teat diameter was 25.41±0.19 mm, ranging from 19.0 mm to 34.0 mm. Correlation analysis showed a significantly (p<0.01) positive correlation between SCC and teat length as well as teat diameter with a correlation coefficient of 0.143 and 0.519, respectively.

## Discussion

### Udder shape and SCC

In the present study, the level of somatic cell count was higher for pendulous shaped udder in comparison of regular shaped udder and the effect of udder shape on degree of intra-mammary infection and SCC level was found significant (p<0.01). The previous studies had also reported a significant effect of udder morphology and stated that cows with pendulous udders had the highest risk of mastitis [12] and higher SCC [13]. Our results are also supported by other studies, where the deeper udders were found to be at higher risk of developing mastitis due to their increased tendency to become soiled [14] and more susceptible to lesions [6-7], hence, being contaminated with environmental pathogens and developed mastitis. However, some study [15] found the insignificant effect of udder shape on SCC and a significant negative correlation between somatic cell number and udder depth has been also reported by Orban *et al*. [16].

### Teat-end shape and SCC

The udder teats are the first line of defense against intra-mammary infection. Present study revealed that flat and inverted teat-end shape may be a risk factor for intra-mammary infection as we found highest SCC level for flat teat-end followed by inverted and least for pointed teat-end and the effect of teat-end shape on SCC was significant (p<0.01). The probability of mastitis occurring varies considerably between different teat shape, sizes, and morphology of the teat-end [17]. There are reports of the significant effect of teat-end shape with higher log SCC value for the inverted teat-end [16]. Non-significant relationship between the SCC and teat-end shape was also reported by Manzi *et al*. [18]. Less pointed and more inverted teat-ends have been associated with increased susceptibility to mastitis as they retain milk, which can act as a substrate for bacterial growth [19]. Hence, it has been suggested that some teat-end shapes act as risk factors for infection, and, as they have high daughter-dam heritability, can be eliminated by selective breeding [20].

### Teat length and teat diameter

In the present study, result pointing out to increase in the degree of intra-mammary infection with an increase in teat length and teat diameter as log SCC was found to be significantly positively correlated with teat morphometry. Similar to present findings, some previous studies also revealed that the occurrence of subclinical mastitis was highest for cows with long and thick teats [7,21] and are a potential risk factor for intra-mammary infection. In contrary to this, few literatures has been showed the higher value of the somatic cell score for shorter teats [4] and a negative correlation of somatic cell number with teat diameter [16]. While, no effect of teat length and teat thickness on SCC have also been reported [22]. Other data suggested machine incompatibilities such as more frequent liner slips and increased likelihood of teat-end lesions in longer teats [23] and in teats with larger diameter [24] which may act as a risk for intra-mammary infections.

## Conclusion

It was concluded from the present study that the pendulous udder, flat and inverted teat-end, very long and thick teat were more susceptible to intra-mammary infection in Jersey crossbred cows. Therefore, these conformation traits must be considered accordingly while selecting dairy animals for future milk production.

## Authors’ Contributions

PB and CB designed the work. PB conducted the study. MAP, MRT, and KPJ helped PB for statistical analysis. PB and SAB prepared the manuscript. PKP revised the manuscript for communication to the journal. All authors read and approved the final manuscript.

## References

[1] Hogeveen H, Huijps K, Lam T (2011). Economic aspects of mastitis:New developments. New Zeal. Vet. J.

[2] Dang A.K, Anand S.K (2007). Effect of milking systems on the milk somatic cell counts and composition. Livest. Res. Rural Dev.

[3] Nakov D, Hristov S, Andonov S, Trajchev M (2014). Udder-related risk factors for clinical mastitis in dairy cows. Vet. Arch.

[4] Nemcova E, Stipkova M, Zavadilova L, Bouska J, Vacek M (2007). The relationship between somatic cell count, milk production and six linearly scored type traits in Holstein cows. Czech J. Anim. Sci.

[5] VanRadeq P.M, Jensen E.L, Lawlor T.L, Funk D.A (1990). Prediction of transmitting abilities for holstein type traits. J. Dairy Sci.

[6] Bhutto A.L, Murray R.D, Woldehiwet Z (2010). Udder shape and teat-end lesions as potential risk factors for high somatic cell counts and intramammary infections in dairy cows. Vet. J.

[7] Singh R.S, Bansal B.K, Gupta D.K (2014). Udder health in relation to udder and teat morphometry in holstein friesian ×Sahiwal crossbred dairy cows. Trop. Anim. Health Prod.

[8] Sharma N, Singh N.K, Bhadwal M.S (2011). Relationship of somatic cell count and mastitis:An overview. Asian-Aust. J. Anim. Sci.

[9] Klein D, Flock M, Khol J.L, Franz S, Stuger H.P, Baumgartner W (2005). Ultrasonographic measurement of the bovine teat:breed differences, and the significance of the measurements for udder health. J. Dairy Res.

[10] Norman H.D, Powell R.L, Wright J.R, Cassell B.G (1988). Phenotypic and genetic relationship between linear functional type traits and milk yield for five breeds. J. Dairy Sci.

[11] SAS Institute Inc (2011). SAS®9.3 System Options:Reference.

[12] Uzmay C, Kaya Y, Akbas Y, Kaya A (2003). Effects of udder and teat morphology, parity and lactation stage on subclinical mastitis in holstein cows. Turk. J. Vet. Anim. Sci.

[13] Ahlawat K, Dang A.K, Singh C (2008). Relationship of teat and udder shape with milk SCC in primiparous and multiparous Sahiwal cows. Indian J. Dairy Sci.

[14] Lopez-Benavides M.G, Williamson J.H, Cursons R.T, Lacy-Hulbert S.J, Woolford M.W, Hogeveen H (2005). *Streptococcus uberis* population dynamics in the New Zealand pastoral dairy farm. Proceedings of the 4^th^ IDF International Mastitis Conference.

[15] Coban O, Sabuncuoglu N, Tuzemen N (2009). A study on relationships between somatic cell count (SCC) and some udder traits in dairy cows. J. Anim. Vet. Adv.

[16] Orban M, Gulyas L, Nemeth S, Gergacz Z (2009). Morphometric evaluation of udders in jersey cows. Sci. Pap. Anim. Sci. Biotechnol.

[17] Bardakcioglu H.E, Sekkin S, Oral Toplu H.D (2011). Relationship between some teat and body measurements of holstein cows and sub-clinical mastitis and milk yield. J. Anim. Vet. Adv.

[18] Manzi M.D.P, Nobrega D.B, Faccioli Y.M, Troncarelli Z, Menozzi B.D, Langoni H (2012). Relationship between teat-end condition, udder cleanliness and bovine subclinical mastitis. Res. Vet. Sci.

[19] Chrystal M.A, Seykora A.J, Hansen L.B, Freeman A.E, Kelley D.H, Healey M.H (2001). Heritability of teat-end shape and the relationship of teat-end shape with somatic cell score for experimental herd of cows. J. Dairy Sci.

[20] Chrystal M.A, Seykora A.J, Hansen L.B (1999). Heritabilities of teat end shape and teat diameter and their relationships with somatic cell score. J. Dairy Sci.

[21] Haghkhah M, Ahmadi M.R, Gheisari H.R, Kadivar A (2011). Preliminary bacterial study on subclinical mastitis and teat condition in dairy herds around Shiraz. Turk. J. Vet. Anim. Sci.

[22] Juozaitiene V, Juozaitis A, Micikeviciene R (2006). Relationship between somatic cell count and milk production or morphological traits of udder in Black and White cows. Turk. J. Vet. Anim. Sci.

[23] Rogers G.W, Hargrove G.L, Lawlor T.J.J, Ebersole J.L (1991). Correlations among linear type traits and somatic cell counts. J. Dairy Sci.

[24] Mein G.A, Reinemann D.J, Schuring N, Ohnstad I (2004). Milking machines and mastitis risk:A storm in a teat-cup.

